# Hepatobiliary risk factors for clinical outcome of Kawasaki disease in children

**DOI:** 10.1186/1471-2431-14-51

**Published:** 2014-02-18

**Authors:** Dae Yong Yi, Ji Young Kim, Eun Young Choi, Jung Yun Choi, Hye Ran Yang

**Affiliations:** 1 Department of Pediatrics, Division of Pediatric Gastroenterology and Hepatology, Seoul National University Bundang Hospital, 166 Gumi-ro, Bundang-gu, Seongnam-si, Gyeonggi-do 463-707, Republic of Korea; 2 Department of Radiology, Seoul National University Bundang Hospital, 166 Gumi-ro, Bundang-gu, Seongnam-si, Gyeonggi-do 463-707, Republic of Korea; 3 Department of Pediatrics, Seoul National University College of Medicine, Seoul, Korea

**Keywords:** Kawasaki disease, Ultrasonography, Acalculous cholecystitis, Coronary artery disease, Child

## Abstract

**Background:**

Kawasaki disease (KD) is an acute febrile vasculitis that causes coronary artery abnormality (CAA) as a complication. In some patients, an association has been noted between elevated liver enzymes or an abnormal gallbladder (GB) and hepatobiliary involvement in KD. In this study, we aimed to evaluate clinical, laboratory, and ultrasonographic (USG) risk factors of hepatobiliary involvement for the intravenous immunoglobulin (IVIG) resistance and the development of CAA in children with KD.

**Methods:**

From March 2004 through January 2013, clinical features, laboratory data, echocardiographic findings, and USG findings were retrospectively reviewed regarding the response to IVIG treatment and coronary artery complications in 67 children with KD. Acute acalculous cholecystitis (AAC) was diagnosed based on USG criteria.

**Results:**

Among all factors, only the prothrombin time international normalized ratio was significantly different between the IVIG-response and IVIG-resistance groups (*p* = 0.024). CAA was statistically more frequent in the AAC group (n = 24) than in the non-AAC group (n = 43) (23.3% vs. 58.3%, *p* = 0.019). Among the laboratory factors, segmented neutrophil percentage, total bilirubin level, and C-reactive protein were significant in children with CAA (*p* = 0.014, *p* = 0.009, and *p* = 0.010). Abnormal GB findings on USG were significantly more frequent in children with CAA than in those without CAA (*p* = 0.007; OR = 4.620; 95% confidence interval [CI]: 1.574–13.558). GB distension on USG was the only significant risk factor for CAA (*p* = 0.001; OR = 7.288; 95% CI: 2.243–23.681) by using multiple logistic regression analysis.

**Conclusion:**

For children in the acute phase of KD, USG findings of the GB, especially GB distension, may be an important risk factor for CAA as a complication.

## Background

Acute acalculous cholecystitis (AAC) is an inflammatory disease of the gallbladder (GB) with symptoms lasting 1 month or less, which was rarely diagnosed in the past but whose incidence is increasing because of increased awareness and improved diagnostic imaging modalities [1,2].

AAC can be diagnosed based on abdominal ultrasonography (USG) findings because of its high specificity within the biliary system and its cost-effectiveness. Although the disease is generally benign and improves with supportive medical treatment, some cases of AAC can require surgical therapy or result in complications, such as septic shock or death, because of underlying diseases or misdiagnosis [3-6]. Thus, early diagnosis and proper treatment are required to reduce morbidity and mortality related to AAC.

Although the etiology and pathogenesis of AAC are as yet unclear, it is known to be associated with concurrent systemic infections, metabolic disorders, and other systemic diseases including Kawasaki disease (KD) [1,4,7].

KD is an acute febrile vasculitis that affects medium-sized arteries; it occurs predominantly in infants and young children [8]. Despite medical treatment, including administration of intravenous immunoglobulin (IVIG), coronary artery abnormality (CAA) is reported to develop as a complication of KD in about 5% of patients [9,10]. The classic diagnosis of KD has generally been based on the presence of fever persisting at least 5 days, changes in the extremities, lips, and oral cavity, polymorphous exanthema, bilateral bulbar conjunctival injection without exudate, and cervical lymphadenopathy, >1.5 cm in diameter and usually unilateral [8]. However, in many patients, the clinical manifestations of KD are atypical and incomplete for definitive diagnosis for KD, which can lead to a delay in diagnosis and possibly a worse prognosis for CAA than occurs with typical KD [11,12]. Thus, rapid suspicion and accurate diagnosis of KD based on clinical manifestations, laboratory studies, and echocardiographic examination, followed by appropriate treatment, may be essential to prevent CAA [8,13].

In some patients with KD, gastrointestinal symptoms such as abdominal pain, nausea, and vomiting or laboratory and radiological hepatobiliary abnormalities can be the initial presentation, masking or overlapping typical symptoms of KD, and sometimes leading to misdiagnosis as a gastrointestinal or hepatobiliary disease such as AAC or hepatitis [10,14,15]. Additionally, there have been only a few studies suggesting the clinical significance of co-existing AAC in patients with KD, and no studies to date have indicated that hepatobiliary involvement may be a risk factor for CAA or IVIG resistance in KD.

Therefore, the present study aimed to analyze and evaluate clinical, laboratory, and USG risk factors for the response to IVIG treatment and the development of CAA as a complication in children with KD.

## Methods

### Patients and data extraction

Children diagnosed with KD and were performed abdominal USG during the acute stage of KD due to hepatobiliary manifestations such as abdominal pain, jaundice, and liver function test abnormalities at the Seoul National University Bundang Hospital from March 2004 to January 2013 were enrolled in the present study. The clinical features, laboratory data, echocardiographic findings, and USG findings were retrospectively reviewed and analyzed, as were coronary artery complications and clinical outcomes related to IVIG treatment. Patients with other systemic inflammation or who did not undergo abdominal USG during the clinical course of KD were excluded from the study.

This study was conducted with the approval from the Institutional Review Board of the Seoul National University Bundang Hospital. Written informed consent was obtained from the patient’s parent for the publication of this report.

### Diagnosis of typical and atypical Kawasaki diseas*e*

Typical KD was diagnosed according to the diagnostic criteria for KD: the presence of fever persisting at least 5 days, plus at least four of the following five diagnostic features, 1) changes in the extremities, 2) changes in the lips and oral cavity, 3) polymorphous exanthema, 4) bilateral bulbar conjunctival injection without exudate, and 5) cervical lymphadenopathy >1.5 cm in diameter, usually unilateral [8].

Atypical KD was diagnosed when there was a high suspicion for KD based on atypical clinical features such as vomiting, diarrhea, abdominal pain, and heart failure, with coronary artery dilatation on echocardiography or more than three supplemental laboratory criteria and with fever persisting for at least 5 days [8,10,12]. The supplemental laboratory criteria are as follows: C-reactive protein (CRP) ≥ 3.0 mg/dL and/or erythrocyte sedimentation rate (ESR) ≥ 40 mm/h with (1) albumin ≤ 3.0 g/dL, (2) anemia for age, (3) elevation of alanine aminotransferase, (4) platelets after 7 days ≥ 450,000/mm^3^, (5) white blood cell (WBC) count ≥ 15,000/mm^3^, and (6) urine WBC ≥ 10 per high-power field [8,10].

### Laboratory investigation

Laboratory data was obtained within 24 hours before initial IVIG administration and included WBC count, percent neutrophils, hemoglobin, platelets, albumin, total bilirubin, direct bilirubin, aspartate aminotransferase, alanine aminotransferase, γ-glutamyl transferase, ESR, CRP, prothrombin time (PT) international normalized ratio (INR), and activated partial thromboplastin time. Hyperbilirubinemia was defined as a serum total bilirubin level exceeding 1.5 mg/dL.

### Radiological evaluation and diagnosis of AAC

For all study subjects, abdominal USG was performed by expert pediatric radiologists during the acute stage of KD, and the sonographic images were reviewed repeatedly by other expert pediatric radiologists.

The US diagnostic criteria for AAC are as follows: (1) GB distention, (2) GB wall thickness more than 3.5 mm, (3) non-shadowing echogenic sludge, and (4) pericholecystic fluid collections [4]. Definite AAC was defined as GB findings on USG satisfying at least two of the four diagnostic criteria for AAC, and suspected AAC was defined as GB findings satisfying at least one criterion. The AAC group was presented as the sum of definite AAC and suspected AAC, and the non-AAC group was defined in patients without any GB abnormalities on abdominal USG.

### Echocardiographic evaluation and definition of CAA and IVIG resistance

CAA was assessed in both the acute phase (within 4 weeks of onset) and the sequelae phase (>4 weeks after onset) by using echocardiography assessed by expert pediatric cardiologists and reviewed repeatedly by other expert pediatric cardiologists. CAA was defined according to the Japanese Ministry of Health criteria [8]. These criteria classify coronary arteries as abnormal when the internal luminal diameter is >3 mm in children aged younger than 5 years and > 4 mm in children older than 5 years; when the internal diameter of a segment measures more than 1.5 times that of an adjacent segment; or when the coronary lumen is clearly irregular [8].

IVIG resistance was defined when persistent or recurrent fever was recorded 36 hours after the completion of initial IVIG administration in patients with KD [14].

### Statistical analysis

Statistical analysis was performed by using SPSS 18.0 statistical software (SPSS Inc., Chicago, IL, USA). All continuous data were presented as medians and ranges. Fisher’s exact test, the Mann–Whitney U test, and logistic regression analysis were applied to evaluate the differences between each group and to determine risk factors. The level of statistical significance was set at *p* < 0.05.

## Results

**Figure 1:**
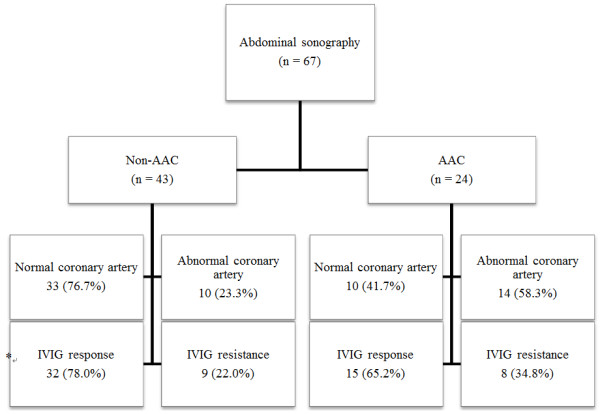
**Coronary artery abnormalities and the response to IVIG therapy were compared according to the gallbladder involvement in children with Kawasaki disease.** AAC: acute acalculous cholecystitis, IVIG: intravenous immunoglobulin. *IVIG was not administered in 2 children of non-AAC group and 1 child of the AAC group.

In total, 67 children with KD (38 boys, 29 girls) underwent abdominal USG during the acute phase of KD; AAC was present in 24 (35.8%) (Figure 1).

Clinical characteristics of patients with KD according to their GB sonographic findings are listed and compared in Table 1. There were no significant differences in age and gender between the 2 groups. The duration of hospitalization was longer in the AAC group, but was not statistically significant (*p* = 0.059). Clinical symptoms such as abdominal pain, vomiting, and duration of fever were not significantly different between the 2 groups. Of the diagnostic criteria for typical KD, changes in the peripheral extremities were significantly more frequent in the AAC group than in the non-AAC group (*p* = 0.021; odds ratio [OR] = 3.714; 95% confidence interval (CI): 1.272–10.847] and conjunctivitis was less frequent in the AAC group (*p* = 0.046; OR = 0.249; 95% CI: 0.064–0.965). There were no significant differences in other diagnostic criteria between the 2 groups.

**Table 1:** Comparison of clinical characteristics of Kawasaki disease according to sonographic findings of gallbladder

**Variables**	**Non AAC group**	**AAC group†**	***P *** **value**
	**(n = 43)**	**(n = 24)**	
Mean age (years)	3.0 (0.2 ~ 10.6)	3.3 (0.8 ~ 7.8)	0.834
Male gender	25 (58.1%)	13 (54.2%)	0.801
Duration of admission (days)	6.0 (3.0 ~ 27.0)	8.0 (4.0 ~ 36.0)	0.059
Abdominal pain/vomiting	24 (55.8%)	13 (54.2%)	1.000
Duration of fever (days)	5.0 (3.0 ~ 9.0)	5.0 (3.0 ~ 19.0)	0.547
Typical Kawasaki disease	23 (53.5%)	16 (66.7%)	0.317
Fever ≥ 5 days	38 (88.4%)	23 (95.8%)	0.408
Dysmorphous skin rash	36 (83.7%)	22 (91.7%)	0.472
Oral mucosal change	33 (76.7%)	19 (79.2%)	0.820
Conjunctivitis	39 (90.7%)	17 (70.8%)	0.046*
Cervical lymphadenopathy	28 (65.1%)	19 (79.2%)	0.228
Peripheral extremity change	17 (39.5%)	17 (70.8%)	0.021*
Duration till recovery of LFT (days)	6.0 (1.0 ~ 43.0)	6.0 (3.0 ~ 100.0)	0.333
Frequency of IVIG infusion	1.0 (0 ~ 2.0)	1.0 (0 ~ 3.0)	0.261

Data are presented as median (range) or numbers (%).

*p < 0.05.

†AAC group: the sum of definite AAC and suspected AAC.

AAC, acute acalculous cholecystitis; IVIG, intravenous immunoglobulin; LFT, liver function test.

Laboratory findings of the patients with KD according to their GB sonographic findings are listed and compared in Table 2. Platelet count and albumin level among laboratory factors were significantly lower in the AAC group than in the non-AAC group (*p* = 0.007 and *p* = 0.001, respectively).

**Table 2:** Comparison of laboratory characteristics of Kawasaki disease according to sonographic findings of gallbladder

**Variables**	**Non AAC group**	**AAC group†**	***P *** **value**
	**(n = 43)**	**(n = 24)**	
WBC (/mm^3^)	13,590 (5,680 ~ 30,300)	13,930 (4,000 ~ 23,620)	0.870
Neutrophils (%)	78.6 (29.3 ~ 95.2)	84.3 (37.5 ~ 95.3)	0.392
Hemoglobin (g/dL)	11.6 (8.7 ~ 14.2)	11.8 (10.0 ~ 14.8)	0.985
Platelet count (/mm^3^)	354,000 (175,000 ~ 573,000)	274,000 (64,000 ~ 492,000)	0.007*
Albumin (g/dL)	3.9 (2.7 ~ 4.6)	3.4 (2.6 ~ 4.3)	0.001*
Total bilirubin (mg/dL)	1.1 (0.2 ~ 5.7)	2.6 (0.2 ~ 5.4)	0.172
Direct bilirubin (mg/dL)	2.1 (0.1 ~ 5.3)	2.4 (1.6 ~ 4.1)	0.704
AST (IU/L)	84 (19 ~ 1,287)	109 (27 ~ 1,003)	0.729
ALT (IU/L)	159 (6 ~ 898)	167 (25 ~ 1,076)	0.870
γGT (IU/L)	219 (23 ~ 577)	189 (20 ~ 450)	1.000
ESR (mm/h)	44.5 (6 ~ 120)	38 (3 ~ 114)	1.000
CRP (mg/dL)	8.0 (0.6 ~ 26.0)	9.4 (0.2 ~ 26.0)	0.392
PT INR	1.2 (1.0 ~ 1.5)	1.2 (1.0 ~ 10.9)	0.460
aPTT (sec)	43.6 (33.2 ~ 58.7)	45.9 (26.8 ~ 57.7)	0.440

Data are presented as median (range).

*p < 0.05.

†AAC group: the sum of definite AAC and suspected AAC.

AAC, acute acalculous cholecystitis; aPTT, activated partial thromboplastin time; ALT, alanine aminotransferase; AST, aspartate aminotransferase; CI, confidence interval; CRP, C-reactive protein; ESR, erythrocyte sedimentation rate; γGT, gamma glutamyl transferase, INR, international normalized ratio; PT, prothrombin time; WBC, white blood cell.

The number of patients with CAA as a complication of KD and the number of patients resistant to IVIG therapy in both the non-AAC group and the AAC group are shown in Figure 1. Out of 67 patients, 3 patients (2 in the non-AAC group and 1 in the AAC group) did not receive IVIG therapy because their fever had subsided before treatment; thus the remaining 64 patients were initially treated with IVIG (2 g/kg/day) and high-dose aspirin (50–100 mg/kg/day). CAAs were more frequent in the AAC group than in the non-AAC group (23.3% vs. 58.3%, *p* = 0.019). Resistance to IVIG therapy was more frequently observed in the AAC group than in the non-AAC group, but the difference was not statistically significant (22.0% vs. 34.8%, *p* = 0.085).

The sonographic and clinical risk factors of IVIG resistance are shown in Table 3. There was no significant difference in abnormal GB findings on USG between the IVIG response group and the IVIG resistance group, nor were there statistically significant differences between the 2 groups in any of the AAC diagnostic criteria. Of the clinical findings, peripheral extremity changes were more frequently observed in the IVIG resistance group, but not significant in OR and 95% CI (*p* = 0.045; OR = 3.579; 95% CI: 0.846–15.136).

**Table 3:** Sonographic and clinical factors for IVIG resistance in children with Kawasaki disease

**Variables**	**IVIG response**	**IVIG resistance**	***P *** **value**	**OR (95% CI)**
	**(n = 47)**	**(n = 17)**		
*Sonographic factors*				
Abnormal GB	15 (31.9%)	8 (47.1%)	0.377	1.896 (0.611 ~ 5.887)
GB distension	10 (21.3%)	8 (47.1%)	0.060	3.289 (1.010 ~ 10.715)
GB wall thickness	5 (10.6%)	0 (0%)	0.313	0.894 (0.810 ~ 0.986)
GB sludge	0 (0%)	2 (11.8%)	0.067	1.133 (0.953 ~ 1.348)
Abnormal liver	11 (23.9%)	2 (11.8%)	0.485	0.424 (0.084 ~ 2.151)
*Clinical factors*				
Hyperbilirubinemia	26 (55.3%)	10 (58.8%)	1.000	1.154 (0.375 ~ 3.551)
Typical Kawasaki disease	28(59.6%)	11(64.7%)	0.778	0.207(0.014 ~ 3.137)
Fever ≥ 5 days	43(91.5%)	16(94.1%)	1.000	0.339(0.012 ~ 9.676)
Dysmorphous skin rash	41(87.2%)	15(88.2%)	1.000	0.647(0.082 ~ 5.093)
Oral mucosal change	37(78.7%)	12(70.6%)	0.517	0.262(0.041 ~ 1.659)
Conjunctivitis	41(87.2%)	13(76.5%)	0.435	0.199(0.023 ~ 1.732)
Cervical lymphadenopathy	34(72.3%)	11(64.7%)	0.552	0.656(0.149 ~ 2.888)
Peripheral extremity change	21(44.7%)	13(76.5%)	0.045*	3.579(0.846 ~ 15.136)

*p < 0.05.

CI, confidence interval; GB, gallbladder; IVIG, intravenous immunoglobulin; OR, odds ratio.

The laboratory risk factors of IVIG resistance are shown in Table 4. Of laboratory factors, PT INR showed significant differences related to IVIG resistance (*p* = 0.024).

**Table 4:** Laboratory factors for IVIG resistance in children with Kawasaki disease

**Variables**	**IVIG response**	**IVIG resistance**	***P *** **value**
	**(n = 47)**	**(n = 17)**	
WBC (/mm^3^)	13,990 (4,000 ~ 28,000)	18,710 (7,460 ~ 30,300)	0.323
Neutrophils (%)	79.1 (29.3 ~ 95.2)	83.9 (63.6 ~ 95.3)	0.342
Hemoglobin (g/dL)	11.6 (9.2 ~ 14.8)	12.2 (8.7 ~ 14.3)	0.460
Platelet count (/mm^3^)	323,000 (64,000 ~ 573,000)	287,000 (94,000 ~ 513,000)	0.861
Albumin (g/dL)	3.8 (2.6 ~ 4.4)	3.7 (3.0 ~ 4.6)	0.801
Total bilirubin (mg/dL)	1.7 (0.2 ~ 5.7)	2.5 (0.6 ~ 4.7)	0.164
Direct bilirubin (mg/dL)	2.1 (0.1 ~ 5.3)	2.2 (1.8 ~ 4.1)	0.595
AST (IU/L)	128 (19 ~ 1,287)	88 (34 ~ 646)	0.837
ALT (IU/L)	172 (6 ~ 1,076)	168 (18 ~ 1,027)	0.677
γGT (IU/L)	227.5 (20 ~ 577)	160.0 (105 ~ 258)	0.365
ESR (mm/h)	38 (3 ~ 120)	47 (30 ~ 72)	0.499
CRP (mg/dL)	8.3 (0.2 ~ 26.0)	10.9 (4.7 ~ 26.0)	0.354
PT INR	1.2 (1.0 ~ 10.9)	1.33 (1.1 ~ 1.5)	0.024*
aPTT (sec)	44.2 (26.8 ~ 58.7)	45.0 (41.8 ~ 55.0)	0.682

Data are presented as median (range).

*p < 0.05.

aPTT, activated partial thromboplastin time; ALT, alanine aminotransferase; AST, aspartate aminotransferase; CI, confidence interval; CRP, C-reactive protein; ESR, erythrocyte sedimentation rate; γGT, gamma glutamyl transferase, INR, international normalized ratio; PT, prothrombin time; WBC, white blood cell.

The sonographic and clinical risk factors of the presence of CAA as a complication of KD are listed in Table 5. Abnormal GB findings on USG were significantly more frequent in the CAA group than in the normal coronary artery group (*p* = 0.007; OR = 4.620; 95% CI: 1.574–13.558). Of the AAC sonographic criteria, GB distension was the only significant risk factor indicating CAA (*p* = 0.001: OR = 7.288: 95% CI: 2.243–23.681), whereas GB wall thickness and GB sludge findings were not risk factors for CAA. Clinically, hyperbilirubinemia was a significant factor for CAA (*p* = 0.009; OR = 4.800; 95% CI: 1.513–15.227).

**Table 5:** Sonographic and clinical factors for coronary artery complications in children with Kawasaki disease

**Variables**	**Normal coronary artery (n = 43)**	**Abnormal coronary artery (n = 24)**	***P *** **value**	**OR (95% CI)**
*Sonographic factors*				
Abnormal GB	10 (23.3%)	14 (58.3%)	0.007*	4.620 (1.574 ~ 13.558)
GB distension	6 (14.0%)	13 (54.2%)	0.001*	7.288 (2.243 ~ 23.681)
GB wall thickness	4 (9.3%)	2 (8.3%)	1.000	0.886 (0.150 ~ 5.235)
GB sludge	2 (4.7%)	0 (0%)	0.533	0.953 (0.893 ~ 1.019)
Abnormal liver	9 (21.4%)	4 (16.7%)	0.755	0.733 (0.199 ~ 2.697)
*Clinical factors*				
Typical Kawasaki disease	22(51.2%)	17(70.8%)	0.132	0.186 (0.011 ~ 3.081)
Fever ≥ 5 days	39(90.7%)	22(91.7%)	1.000	0.241 (0.012 ~ 4.910)
Dysmorphous skin rash	36(83.7%)	22(91.7%)	0.472	1.711 (0.241 ~ 12.164)
Oral mucosal change	32(74.4%)	20(83.3%)	0.545	0.832 (0.145 ~ 4.768)
Conjunctivitis	37(86.0%)	19(79.2%)	0.505	0.144 (0.012 ~ 1.725)
Cervical lymphadenopathy	27(62.8%)	20(83.3%)	0.099	2.504 (0.558 ~ 11.225)
Peripheral extremity change	20(46.5%)	14(58.3%)	0.447	1.498 (0.444 ~ 5.055)
IVIG resistance	10 (24.4%)	7 (30.4%)	0.769	1.356 (0.434 ~ 4.236)
Hyperbilirubinemia	19 (44.2%)	19 (79.2%)	0.009*	4.800 (1.513 ~ 15.227)

*p < 0.05.

CI, confidence interval; GB, gallbladder, IVIG, intravenous immunoglobulin; OR, odds ratio.

The laboratory factors of CAA as a complication of KD are listed in Table 6. Among laboratory factors, segmented neutrophil percentage, total bilirubin level, and CRP were significant in CAA group (*p* = 0.014, *p* = 0.009, and *p* = 0.010, respectively).

**Table 6:** Laboratory factors for coronary artery complications in children with Kawasaki disease

**Variables**	**Normal coronary artery**	**Abnormal coronary artery**	***P *** **value**
	**(n = 43)**	**(n = 24)**	
WBC (/mm^3^)	13,010 (4,000 ~ 30,300)	14,435 (5,020 ~ 25,740)	0.172
Neutrophils (%)	76.6 (29.3 ~ 91.0)	85.4 (45.9 ~ 95.3)	0.014*
Hemoglobin (g/dL)	11.6 (8.7 ~ 14.3)	11.7 (10.0 ~ 14.8)	0.694
Platelet count (/mm^3^)	333,000 (94,000 ~ 573,000)	303,000 (64,000 ~ 462,000)	0.556
Albumin (g/dL)	3.9 (2.6 ~ 4.6)	3.6 (2.7 ~ 4.4)	0.060
Total bilirubin (mg/dL)	1.0 (0.2 ~ 4.7)	2.5 (0.4 ~ 5.7)	0.009*
Direct bilirubin (mg/dL)	2.2 (0.1 ~ 4.1)	2.0 (0.8 ~ 5.3)	0.964
AST (IU/L)	84.0 (19 ~ 1,287)	128.5 (27 ~ 646)	0.565
ALT (IU/L)	168.0 (6 ~ 1,076)	146.5 (28 ~ 1,027)	0.501
γGT (IU/L)	222.5 (20 ~ 577)	187.5 (89 ~ 525)	0.981
ESR (mm/h)	40 (6 ~ 120)	72 (3 ~ 114)	0.328
CRP (mg/dL)	7.4 (0.6 ~ 26.0)	11.1 (0.2 ~ 26.0)	0.010*
PT INR	1.2 (1.0 ~ 1.5)	1.2(1.0 ~ 10.9)	0.839
aPTT (sec)	45.0 (33.7 ~ 58.7)	44.2 (26.8 ~ 57.7)	0.603

Data are presented as median (range).

*p < 0.05.

aPTT, activated partial thromboplastin time; ALT, alanine aminotransferase; AST, aspartate aminotransferase; CI, confidence interval; CRP, C-reactive protein; ESR, erythrocyte sedimentation rate; γGT, gamma glutamyl transferase, INR, international normalized ratio; PT, prothrombin time; WBC, white blood cell.

In multiple logistic regression analysis, GB distension was the only significant risk factors for CAA, out of all the clinical, laboratory and sonographic factors (*p* = 0.001; OR = 7.288; 95% CI: 2.243–23.681).

## Discussion

In patients with KD, hepatobiliary manifestations such as right upper quadrant abdominal pain or jaundice accompanied by persistent fever are relatively frequent atypical symptoms, making the diagnosis obscure when these manifest as the initial major symptoms. The difficulty in diagnosing KD with atypical manifestations may cause a delay in appropriate treatment during the acute phase and thus may increase the risk for complications, including CAA. Therefore, in this study, we tried to determine significant clinical, laboratory, or ultrasonographic factors for IVIG resistance and the development of CAA in children with KD.

The most common etiology of acute cholecystitis in adults and chronic cholecystitis in children is gallstones, for which surgical treatment can be considered. However, medical treatment rather than surgical management should be preferentially considered in pediatric patients with AAC, and disease progression, or ongoing complications in addition to AAC, should be assessed through laboratory tests and abdominal USG.

Though there are differences in accordance with studies, GB abnormality in KD patients occurs 15% during the first 2 weeks of the illness [8]. However, the prevalence is expected to increase with development of USG and concern about association with KD. The etiology of GB abnormalities in patients with KD, such as an increase in GB wall thickness or the GB distension observed in AAC, is unclear; however, several hypotheses have been proposed. Because KD is a systemic vasculitis, it has been suggested that vasculitis of the gastrointestinal organs, including the liver and biliary tract, could be one of the etiologies [14,16]. Another study reported that the gastrointestinal tract in KD is the primary site for the entry of etiologic agents that predispose to KD [14,17]. Yet another study suggested adenopathy around the cystic duct causing obstruction, a secondary vasculitic process of the GB wall, and inflammatory infiltrates with polymorphs, lymphocytes, and eosinophils as possible mechanisms [4,18,19], although the exact etiology remains unclear.

The etiology of LFT abnormality in KD patients is also not clear yet, but some hypotheses were proposed; generalized inflammation, vasculitis, congestive heart failure secondary to myocarditis, nonsteroidal anti-inflammatory antipyretics, toxin-mediated effects, or a combination of these events [19]. Serum transaminase levels were elevated in less than 40% of KD patients, serum bilirubin level in 10%, γ-glutamyl transferase in approximately 67%, and hypoalbuminemia was common associated with more severe and prolonged clinical courses [8].

Hepatobiliary complications may not be a major cause of mortality in patients with KD. However, AAC related to KD can delay the diagnosis of KD, due to the vagueness of the clinical manifestations, and thus may increase the risk for coronary artery complications. Previous studies have shown that incomplete clinical manifestations seemed to be associated with an increased risk for CAA [11,13,20,21]. Hepatobiliary manifestations such as AAC must therefore be carefully assessed in every patient with atypical symptoms that are suspicious for KD.

The diagnosis of KD based on laboratory tests is not easy because the laboratory diagnostic criteria of KD are incomplete and not specific. However, several laboratory findings along with clinical features such as Harada score may give more information for diagnosis and be used to determine risk factors of KD [8]. In the present study, platelet count and serum albumin level suggested in the Harada score were lower in the AAC group.

The prognosis of KD can be considered from the aspects of clinical response to IVIG treatment and ongoing complications of the coronary arteries. With reference to laboratory factors indicating IVIG resistance, PT INR was the only factors of IVIG resistance of those we tested. The other laboratory findings did not show any clinical significance. In previous studies, CRP and total bilirubin at admission were suggested as significant predictors for IVIG resistance, and pre-IVIG treatment serum albumin levels was also noted to be a useful predictor of IVIG resistance in patients with KD [19,22], although these laboratory factors were not significant in our study. Additionally, regarding response to IVIG treatment, Chen et al. [18] reported that sonographic GB abnormalities were associated with IVIG resistance in KD. However, in our study, abnormal GB findings on USG were also not a significant risk factor for IVIG resistance in children with KD.

With regard to clinical and laboratory risk factors for the development of CAA in patients with KD, we found that higher values for segmented neutrophil percentage, total bilirubin levels, and CRP were significantly related to CAA in our study. According to Song et al. [11], incomplete clinical manifestations in patients aged <1 year and IVIG resistance along with Harada score in 10 patients aged > 5 years were associated with increased risk for CAA. In Song’s study, WBC count, CRP, and low serum albumin levels (part of the Harada score), ESR, total bilirubin levels, and low sodium levels were related to the risk of developing CAA, as was suggested by our study results, but were not significant on performing multivariable logistic regression analysis. The other studies revealed that there were several atypical clinical and laboratory findings in patients with KD with CAA, although it was difficult to identify a suitable predictive marker [20,21]. Therefore, it may be worth recommending that KD patients with increases in segmented neutrophil percentage, total bilirubin levels, and CRP should be examined closely for the occurrence of CAA as a significant complication of KD, although there might be some limitations in our study.

As for sonographic risk factors for CAA in patients with KD, there have been few studies reported before ours. Interestingly, we found that in patients with KD, AAC was significantly associated with increased risk for CAA, especially if GB distension was present on abdominal USG. By using multiple logistic regression analysis, GB distension was the only significant variable that related the occurrence of CAA in the present study. Recently, Chen et al. [18] reported that GB abnormalities such as AAC or GB hydrops were merely related to IVIG resistance, and the studies on the relationship between GB findings and coronary complications of KD are rare. In the present study, we could confirm that AAC on abdominal USG, especially with GB distension, was a meaningful finding that was associated with the development of CAA in patients with KD by using both simple and multiple logistic regression analysis. Therefore, more intensive management may be recommended for patients with KD with hepatobiliary manifestations, particularly when GB distension is seen on USG, and findings are compatible with incomplete KD diagnostic criteria.

There are some limitations to our study. Because we enrolled only patients with KD who underwent abdominal USG, this study might not have reflected the clinical and laboratory findings of patients who did not undergo USG; we also might not have enrolled patients who may have had AAC or GB distension that naturally improved. A further well-designed prospective study on a larger scale may be required in the future to overcome these limitations.

## Conclusion

In conclusion, USG findings of the GB in the acute phase of KD, especially the presence of GB distension, might be an important risk factor for CAA as a complication. Thorough USG investigation of the GB should be considered in children with KD who have clinical symptoms and laboratory findings suggesting hepatobiliary involvement of KD, such as abdominal pain, jaundice, and hyperbilirubinemia, to detect GB distension on USG and to diagnose AAC, which requires more intensive treatment.

## Abbreviations

AAC: Acute acalculous cholecystitis; GB: Gallbladder; USG: Ultrasonography; KD: Kawasaki Disease; IVIG: Intravenous immunoglobulin; CAA: Coronary artery abnormality; CRP: C-reactive protein; ESR: Erythrocyte sedimentation rate; WBC: White blood cell; PT: Prothrombin time; INR: International normalized ratio.

## Competing interests

The authors declare that they have no competing interests.

## Authors’ contributions

DYY and HRY designed the study, analyzed the data, and wrote the manuscript. JYK performed UGS and reviewed the results. EYC and JYC performed echocardiography and reviewed the results. All authors have read and approved the final manuscript.

## Pre-publication history

The pre-publication history for this paper can be accessed here:

http://www.biomedcentral.com/1471-2431/14/51/prepub
